# Supplementary material for: Rendering protein structures inside cells at the atomic level with Unreal Engine
Source: bioRxiv. 2024 Mar 9:2023.12.08.570879. Preprint. [Version 2] doi: 10.1101/2023.12.08.570879 (PMC10942390; doi:10.1101/2023.12.08.570879)
Supplement: 1 [file NIHPP2023.12.08.570879V2-supplement-1.pdf]

bioRxiv preprint doi: https://doi.org/10.1101/2023.12.08.570879; this version posted March 9, 2024. The copyright holder for this preprint (which was not certified by peer review) is the author/funder, who has granted bioRxiv a license to display the preprint in perpetuity. It is made available under aCC-BY 4.0 International license.

Supplementary table 1.
Protein structures shown in the P22 infecting bacteria scene

Macromolecule name	PDB ID
tRNA	7k00
Ribosome	7k00
P22 tail spike	1tyx
P22 portal	1tyx
P22 ejection mechanism	N/A
P22 bacteriophage	5uu5
Hsp70/DnaK	2kho
GroES	1pcq
GroEL	5w0s
ATP synthase	6oqr
Cell wall	N/A
Bacterial lipoproteins	1eq7
Efflux pump	5o66
Bacteria flagella	7cbl
NADH dehydrogenase	4nwz
Outer membrane protein A	AF-P0A910
Outer membrane protein C	7jz3
Type IVa pilus	3jc8
Respiratory complex I	3m9s
Type III secretion system	7ah9
Ubiquinol oxidase	1fft

bioRxiv preprint doi: https://doi.org/10.1101/2023.12.08.570879; this version posted March 9, 2024. The copyright holder for this preprint (which was not certified by peer review) is the author/funder, who has granted bioRxiv a license to display the preprint in perpetuity. It is made available under aCC-BY 4.0 International license.

DNA (B-form)	1bna
Bacteria inner membrane	CHARMM
Bacteria outer membrane	CHARMM
DNA template	7mkd
RNA polymerase	7mkd
DNA template	6ztj
Bacterial Expressome	6ztj
Enolase	1e9i
Phosphoglycerate kinase	1zmr
Glycerol-3-phosphate dehydrogenase	3da1
HtpG (bacterial Hsp90)	2ioq
Glyceraldehyde 3-phosphate dehydrogenase	6utn
Glycerol kinase	1bo5
Malate dehydrogenase	2pwz
